# The causal effect of Alzheimer’s disease and family history of Alzheimer’s disease on non-ischemic cardiomyopathy and left ventricular structure and function: a Mendelian randomization study

**DOI:** 10.3389/fgene.2024.1379865

**Published:** 2024-06-05

**Authors:** Zhenjie Li, Xiandong Chen, Wangping He, Huazeng Chen, Dehai Chen

**Affiliations:** Department of Cardiovascular Surgery, The First People’s Hospital of Zhaoqing, The First Affiliated Hospital of Zhaoqing Medical College, Zhaoqing, China

**Keywords:** Alzheimer’s disease, non-ischemic cardiomyopathy, dilated cardiomyopathy, left ventricular structure and function, Mendelian randomization

## Abstract

**Background:**

Previous studies have shown that Alzheimer’s disease (AD) can cause myocardial damage. However, whether there is a causal association between AD and non-ischemic cardiomyopathy (NICM) remains unclear. Using a comprehensive two-sample Mendelian randomization (MR) method, we aimed to determine whether AD and family history of AD (FHAD) affect left ventricular (LV) structure and function and lead to NICM, including hypertrophic cardiomyopathy (HCM) and dilated cardiomyopathy (DCM).

**Methods:**

The summary statistics for exposures [AD, paternal history of AD (PH-AD), and maternal history of AD (MH-AD)] and outcomes (NICM, HCM, DCM, and LV traits) were obtained from the large European genome-wide association studies. The causal effects were estimated using inverse variance weighted, MR-Egger, and weighted median methods. Sensitivity analyses were conducted, including Cochran’s Q test, MR-Egger intercept test, MR pleiotropy residual sum and outlier, MR Steiger test, leave-one-out analysis, and the funnel plot.

**Results:**

Genetically predicted AD was associated with a lower risk of NICM [odds ratio (OR) 0.9306, 95% confidence interval (CI) 0.8825–0.9813, *p* = 0.0078], DCM (OR 0.8666, 95% CI 0.7752–0.9689, *p* = 0.0119), and LV remodeling index (OR 0.9969, 95% CI 0.9940–0.9998, *p* = 0.0337). Moreover, genetically predicted PH-AD was associated with a decreased risk of NICM (OR 0.8924, 95% CI 0.8332–0.9557, *p* = 0.0011). MH-AD was also strongly associated with a decreased risk of NICM (OR 0.8958, 95% CI 0.8449–0.9498, *p* = 0.0002). Different methods of sensitivity analysis demonstrated the robustness of the results.

**Conclusion:**

Our study revealed that AD and FHAD were associated with a decreased risk of NICM, providing a new genetic perspective on the pathogenesis of NICM.

## 1 Introduction

Non-ischemic cardiomyopathy (NICM) refers to a group of myocardial diseases that are not caused by coronary artery disease or ischemic injury. NICM has numerous etiologies and leads to high mortality rates, making it the most common cause of non-ischemic systolic heart failure (HF) (Elliott et al., 2008; Pimentel et al., 2016). It was reported that about 30% of patients with NICM die from sudden cardiac death (Kusumoto et al., 2014). NICM includes several subtypes, including hypertrophic cardiomyopathy (HCM), dilated cardiomyopathy (DCM), and restrictive cardiomyopathy (RCM) (Elliott et al., 2008). HCM and DCM are the most commonly diagnosed forms of NICM in clinical practice, with an estimated prevalence of 1/200 and 1/250–2500 among adults, respectively (Wang et al., 2023). Although the exact etiology of NICM is not fully understood, a hereditary component is known to be involved. HCM, an autosomal dominant cardiomyopathy, is characterized by an asymmetric increase in the thickness of the ventricular wall. Approximately 60% of patients with HCM are caused by genetic mutations related to sarcomeric genes. Other genetic diseases, such as mitochondrial diseases, metabolic disorders, and Fabry’s disease, can also lead to HCM. In addition, cardiac amyloidosis may contribute to the development of HCM by acting as an intermediate phenotype (Wang et al., 2023). DCM, on the other hand, is characterized by left ventricular diastolic dysfunction and poor ventricular remodeling (Japp et al., 2016). It is estimated that 30%–48% of DCM cases are hereditary (Weintraub et al., 2017). Genetic factors may also play a role in the pathogenesis of DCM in patients with chemotherapy-related cardiac dysfunction or alcohol intolerance (Wang et al., 2023). Further studies are needed to better understand the underlying causes of NICM and develop effective treatments for this devastating condition.

Alzheimer’s disease (AD), a progressive neurodegenerative disorder that accounts for about 70% of all cases of dementia, has also been implicated in the development of cardiomyopathy (Tublin et al., 2019). The risk of AD is 60%–80% depending on heritable factors and is characterized by the presence of β-amyloid (Aβ) plaques and tau neural fibrillary tangles (Scheltens et al., 2021). Genetic factors commonly associated with AD, such as apolipoprotein E (apoE) 4 and presenilin mutations, were also found to be associated with HF and DCM, suggesting an underlying genetic link between AD and cardiovascular diseases (Tublin et al., 2019). Moreover, misfolded proteins play a key role not only in AD but also in cardiac hypertrophy and cardiomyopathy (Willis and Patterson, 2013). Studies on the relationship between plasma Aβ40 and cardiac function have shown that Aβ40 is associated with decreased left ventricular end-systolic volume (LVESV), LV stroke volume index, and VO2 Max, in the absence of overt cardiovascular diseases (Stamatelopoulos et al., 2018).

Although traditional observational studies have suggested a link between Alzheimer’s disease (AD) and cardiomyopathy, the causal relationship remains uncertain. Mendelian randomization (MR) analysis offers a potential solution by using genetic variations. It uses single nucleotide polymorphisms (SNPs), from genome-wide association studies (GWAS) as instrumental variables (IVs) for environmental exposure. This approach can help clarify the causal relationship between exposure and outcome (Lawlor et al., 2008). Given that genetic factors play a significant role in the pathogenesis of AD (Scheltens et al., 2021), a two-sample MR study was conducted to investigate the causal effects of AD and family history of AD (FHAD) on NICM and LV structure and function.

## 2 Materials and methods

### 2.1 Study design

**FIGURE 1:**
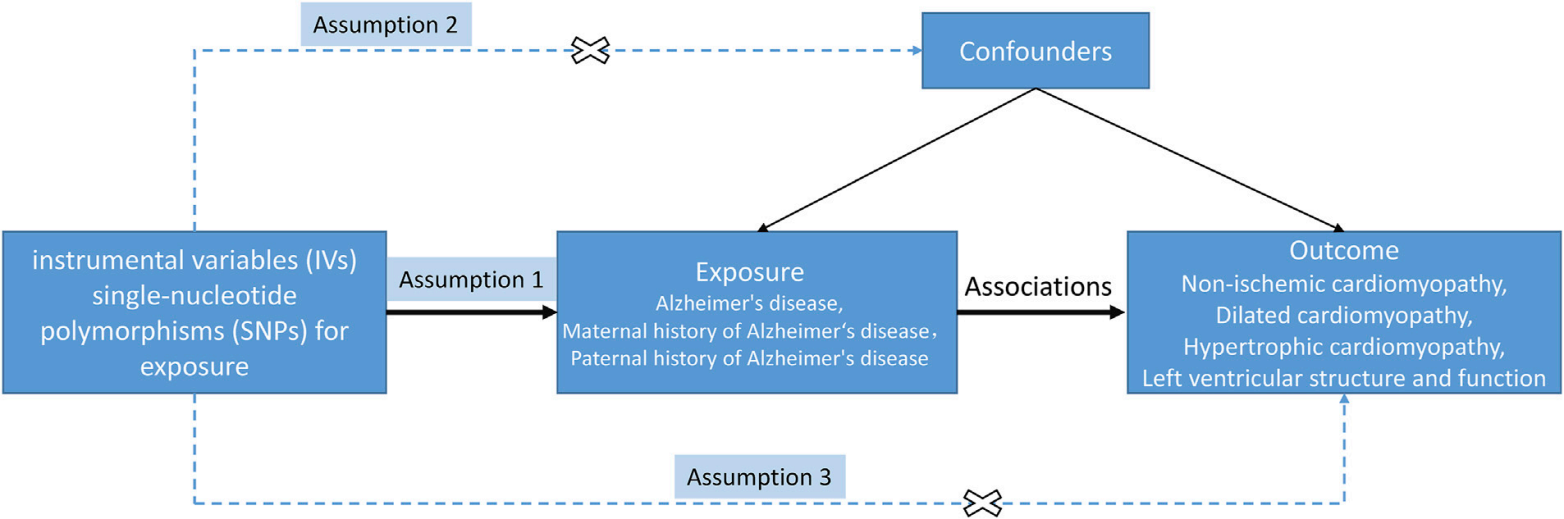
Study design of MR between AD (family history included) and NICM, and left ventricular structure and function. The solid/dash lines indicate relevance/irrelevance. Dash lines with a cross means that instrumental variants (IVs) were independent of confounders (assumption 2) and IVs only affected the outcome via exposure but not via other vicegerent routes (assumption 3).

A two-sample MR analysis was conducted to investigate the causal effects of AD and FHAD on NICM and LV structure and function (Figure 1). The MR design satisfied three key assumptions: 1) the instrumental variables (IVs) were strongly associated with the exposure; 2) IVs were independent of confounding factors on the exposure-outcome pathway; and 3) IVs could only be associated with the outcomes through exposure, not through other pathways (Lawlor, 2016). In this MR analysis, three AD-related traits were used as exposures: AD itself, paternal history of AD (PH-AD), and maternal history of AD (MH-AD). NICM, HCM, and DCM were the three types of cardiomyopathy considered as outcomes. Five structural and functional parameters were the LV traits measured in this study: LV end-diastolic volume (LVEDV), LVESV, ejection fraction (LVEF), mass (LVM), and left ventricular mass-to-end-diastolic volume ratio (LVMVR, also known as LV remodeling index) (Buakhamsri et al., 2009; Tadros et al., 2021).

### 2.2 Data resource

GWAS summary statistics for the outcomes of non-ischemic cardiomyopathy (NICM) and hypertrophic cardiomyopathy (HCM) were obtained from the FinnGen research project. The FinnGen project provided GWAS-level data on 11,400 cases of NICM and 175,752 control subjects, and 556 cases of HCM and 218,236 control subjects (Kurki et al., 2023). For the genetic predictors of dilated cardiomyopathy (DCM), data were extracted from a GWAS conducted on European ancestry. This study included 1,444 cases and 353,937 controls (Sakaue et al., 2021). Additionally, GWAS data on five LV traits (LVEDV, LVESV, LVEF, LVM, and LVMVR) were derived from cardiac magnetic resonance (CMR) imaging of 19,260 UK Biobank participants. The mean age of these participants was 63 ± 8 years, with 46% being male. Exclusion criteria were uncontrolled hypertension and extreme body mass indexes. None of the participants had heart failure, cardiomyopathy, myocardial infarction, or structural heart disease at the time of CMR imaging (Tadros et al., 2021).

Genetic instruments for AD were derived from the UK Biobank, including 39,106 cases and 46,828 controls (Bellenguez et al., 2022). In addition, genetic instruments for FHAD, including paternal history of AD (PH-AD) and maternal history of AD (MH-AD), were derived from a UK Biobank dataset. This dataset included 314,278 participants, with 27,696 maternal cases and 14,338 paternal cases (Bellenguez et al., 2022). To ensure the reliability of the results, a different GWAS dataset (AD-DD) published in 2019, was used to repeat the MR analysis. The AD-DD dataset was extracted from the Alzheimer Disease Genetics Consortium and included 21,982 cases and 41,944 controls (Kunkle et al., 2019). In addition to these datasets, genetic instruments for FHAD, including PH-AD and MH-AD, were extracted from the same UK Biobank dataset. This dataset included 314,278 participants, with 27,696 maternal cases and 14,338 paternal cases (Marioni et al., 2018). All participants in these datasets were of European origin. A summary description of the GWAS datasets is provided in Table 1.

**TABLE 1:** Summary of the GWAS included in the current MR study.

Traits	N.case	Control	Sample size	N.SNPs	Year	Data source
AD	39,106	46,828	85,934	20,921,626	2022	PMID: 35379992
AD-DD	21,982	41,944	63,926	10,528,610	2019	PMID: 30820047
MH-AD	27,696	260,980	288,676	7,776,415	2018	PMID: 29777097
PH-AD	14,338	245,941	260,279	7,776,415	2018	PMID: 29777097
NICM	11,400	175,752	187,152	16,380,358	2021	FinnGen
DCM	1,444	353,937	355,381	19,080,278	2021	PMID: 34594039
HCM	556	218,236	207,011	16,380,466	2021	FinnGen
LV traits	—	—	19,260	9,837,886	2021	PMID: 33495596

AD, Alzheimer’s disease; AD-DD, Different dataset for Alzheimer’s disease; DCM, dilated cardiomyopathy; GWAS, genome-wide association study; HCM, hypertrophic cardiomyopathy; LV, left ventricular; MH-AD, maternal history of Alzheimer’s disease; NICM, non-ischemic cardiomyopathy; N.cases, the numbers of cases; N.SNPs, the numbers of single nucleotide polymorphisms; PH-AD, Paternal history of Alzheimer’s disease.

### 2.3 Instrumental variables selection

SNPs genetically associated with exposures at the genome-wide significance level of *p* < 5 × 10^−8^ were selected as IVs, and linkage disequilibrium (LD) was measured using *r* ^*2*^ < 0.001 and LD distance >10,000 kb (Clarke et al., 2012). Then, we calculated the F statistic to distinguish weak instrumental bias (*F* < 10) (Burgess and Thompson, 2011). The correlation hypothesis was evaluated using the *F* statistic: *F* = *R* ^*2*^(N-1-K)/(1-*R* ^*2*^)K, where *R* ^*2*^ reflects the proportion of SNPs in explaining the exposure variable, N is the sample size, and K is the number of SNPs (Davies et al., 2018). Following the MR assumptions mentioned above, we extracted and screened SNPs associated with corresponding exposures and confounders from the Open GWAS catalog (https://www.ebi.ac.uk/gwas/) and PhenoScanner V2 database (Kamat et al., 2019).

### 2.4 Mendelian randomization analyses

#### 2.4.1 Estimation of causal association

Three MR methods were used to investigate the causal effects of exposures on outcomes. The inverse variance weighted (IVW) method was used as the main MR method to determine the MR estimate for each exposure. The IVW method can be used for meta-analyses to combine the causal effects of SNPs on the outcome with the Wald ratio in the absence of horizontal pleiotropy (Burgess et al., 2016). A random-effects IVW model was adopted in the presence of heterogeneity. The other two methods, including the MR-Egger method (Bowden et al., 2015) and the weighted median (WM) method (Bowden et al., 2016), helped provide the consistency of the direction of MR results. The MR-Egger method and weighted median (WM) method were used to obtain reliable estimates. The latter can provide a robust estimate when more than half of the SNPs satisfy the IV assumptions (Bowden et al., 2016). The consistency of the direction of MR results suggests the solidness of the MR results. The causal estimates are presented as odds ratios (ORs) and 95% confidence intervals (CIs). The Bonferroni correction was executed to correct *p*-values in the presence of multiple tests (Armstrong, 2014). A *p*-value less than 7.82 × 10^−4^ (0.05/64) was considered strong evidence of a causal association. Correlation with the original *p*-value less than 0.05 was considered a nominal causal association.

#### 2.4.2 Sensitivity analysis

For associations with a *p*-value less than 0.05 in the IVW method, sensitivity analyses were performed to assess the robustness of the primary causal estimate. The MR-Egger method was used to detect directional pleiotropy indicated by the intercept. A *p*-value of more than 0.05 indicated the absence of directional pleiotropy; however, it was not sensitive to outliers (Bowden et al., 2016). The MR-multi-directional residual sum and outliers (MR-PRESSO) method was employed to efficiently identify and address outlier SNPs and potential horizontal pleiotropy (Verbanck et al., 2018). The leave-one-out test was used to assess the stability of MR results by excluding SNPs one by one. The Cochran’s Q statistic was adopted to evaluate the heterogeneity for the IVW model (Bowden et al., 2019). Moreover, the MR Steiger directionality test was used to assess the accuracy of the direction of the causal association between exposures and outcomes (Hemani et al., 2017). All analyses were conducted using the TwoSampleMR (version 0.5.6) and MR-PRESSO (version 1.0) packages of R Studio version 4.3.0.

## 3 Results

### 3.1 Selection of instrumental variables

The MR analysis of the causal effects of AD/AD-DD/PH-AD/MH-AD on NICM/DCM/HCM indicated 56/59/56 IVs (AD:NICM/DCM/HCM), 18/18/18 IVs (AD-DD:NICM/DCM/HCM), 3/3/3 IVs (PH-AD:NICM/DCM/HCM), and 4/4/4 IVs (MH-AD:NICM/DCM/HCM). For the causal effects of AD on five LV traits, MR analysis found 58/58/58/57 IVs (AD:LVEDV/LVESV/LVEF/LVM/LVMVR). SNPs explained 1.0151% (AD:NICM), 1.0259% (AD:DCM), 0.7044% (AD-DD:DCM), 0.4645% (PH-AD:NICM), 1.0412% (MH-AD:NICM), and 5.8094% (AD:LVMVR) of the variance. Specific information about IVs with significant associations is provided in Supplementary Tables S1–S5. All the *F* statistics for genetic instruments were >10, indicating no evidence of weak instrument bias.

### 3.2 Mendelian randomization analyses

#### 3.2.1 The causal effect of genetically predicted AD on NICM/DCM/HCM

**FIGURE 2:**
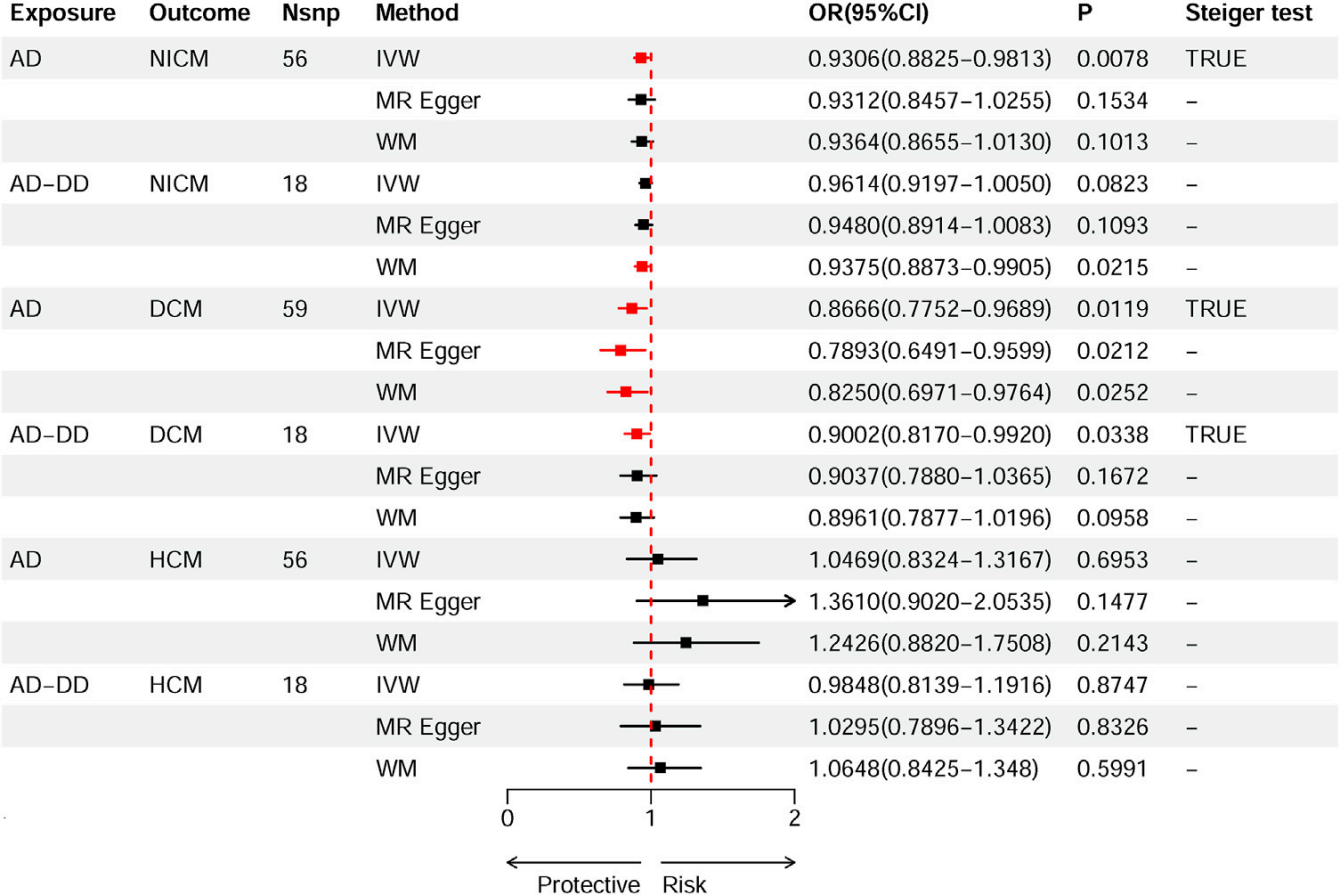
Causal effects of AD on NICM, DCM, and HCM. Summary of the Mendelian randomization (MR) estimates derived from the inverse variance weighted (IVW), weighted median (WM), and MR-Egger methods. AD, Alzheimer’s disease; AD-DD, different dataset for Alzheimer’s disease; NICM, non-ischemic cardiomyopathy; DCM, dilated cardiomyopathy; HCM, hypertrophic cardiomyopathy.

According to the standard IVW method, we estimated the causal effects of AD on NICM/DCM/HCM. Based on the IVW estimates, the effects of SNPs (ORs) genetically associated with AD on NICM, DCM, and HCM were 0.9306 (95% CI 0.8825–0.9813, *p* = 0.0078), 0.8666 (95% CI 0.7752–0.9689, *p* = 0.0119), and 1.0469 (95% CI 0.8324–1.3167, *p* = 0.6953), respectively (Figure 2). In addition, other MR methods, including MR-Egger method and WM method, yielded consistent direction of the results except for the causal estimate between AD and HCM. Using another dataset of AD (AD-DD) on DCM, the IVW model showed similar results (OR 0.9002, 95% CI 0.8170–0.9920, *p* = 0.0338). The results provided suggestive evidence for the effect of AD on NICM/DCM when referring to our preset statistically significant threshold (*p* < 7.82 × 10^−4^). The scatter plots depicting the effect of AD on NICM/DCM are shown in Figure 5. The details can be obtained in Supplementary Table S6.

#### 3.2.2 The causal effect of genetically predicted family history of AD on NICM/DCM/HCM

**FIGURE 3:**
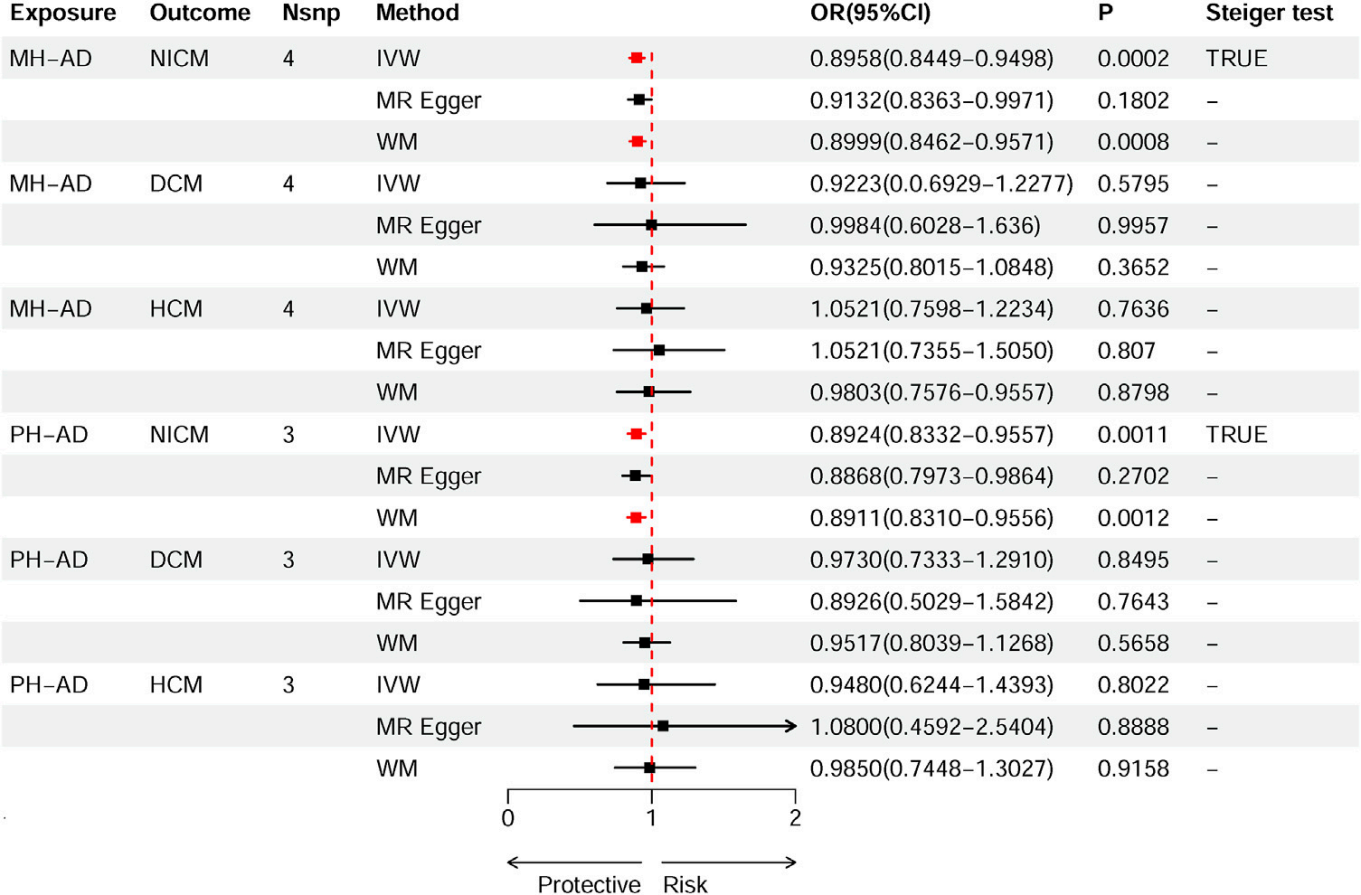
Causal effects of MH-AD on NICM, DCM, and HCM. Summary of the Mendelian randomization (MR) estimates derived from the inverse variance weighted (IVW), weighted median (WM), and MR-Egger methods. AD, Alzheimer’s disease; AD-DD, different dataset for Alzheimer’s disease; DCM, dilated cardiomyopathy; HCM, hypertrophic cardiomyopathy; MH-AD, maternal history of Alzheimer’s disease; NICM, non-ischemic cardiomyopathy; PH-AD, paternal history of Alzheimer’s disease.

Based on IVW estimates, genetically predicted PH-AD was nominally associated with a decreased risk of NICM (OR 0.8924, 95% CI 0.8332–0.9557, *p* = 0.0011), and genetically predicted MH-AD was strongly associated with a decreased risk of NICM (OR 0.8958, 95% CI 0.8449–0.9498, *p* = 0.0002) (Figure 3). We found no causal effect of genetically predicted family history of AD on DCM (PH-AD: OR 0.9730, 95% CI 0.7333–1.2910, *p-*_*IVW*_ = 0.8495; MH-AD: OR 0.9223, 95% CI 0.6929–1.2277, *p-*_*IVW*_ = 0.5795) or HCM (PH-AD: OR 0.9480, 95% CI 0.6244–1.4393, *p-*_*IVW*_ = 0.8022; MH-AD: OR 1.0521, 95% CI 0.7598–1.2234, *p-*_*IVW*_ = 0.7636). The scatter plots for the effect of PH-AD/MH-AD on NICM are shown in Figure 5. The details are provided in Supplementary Table S7.

#### 3.2.3 The causal effect of genetically predicted AD on LV traits

**FIGURE 4:**
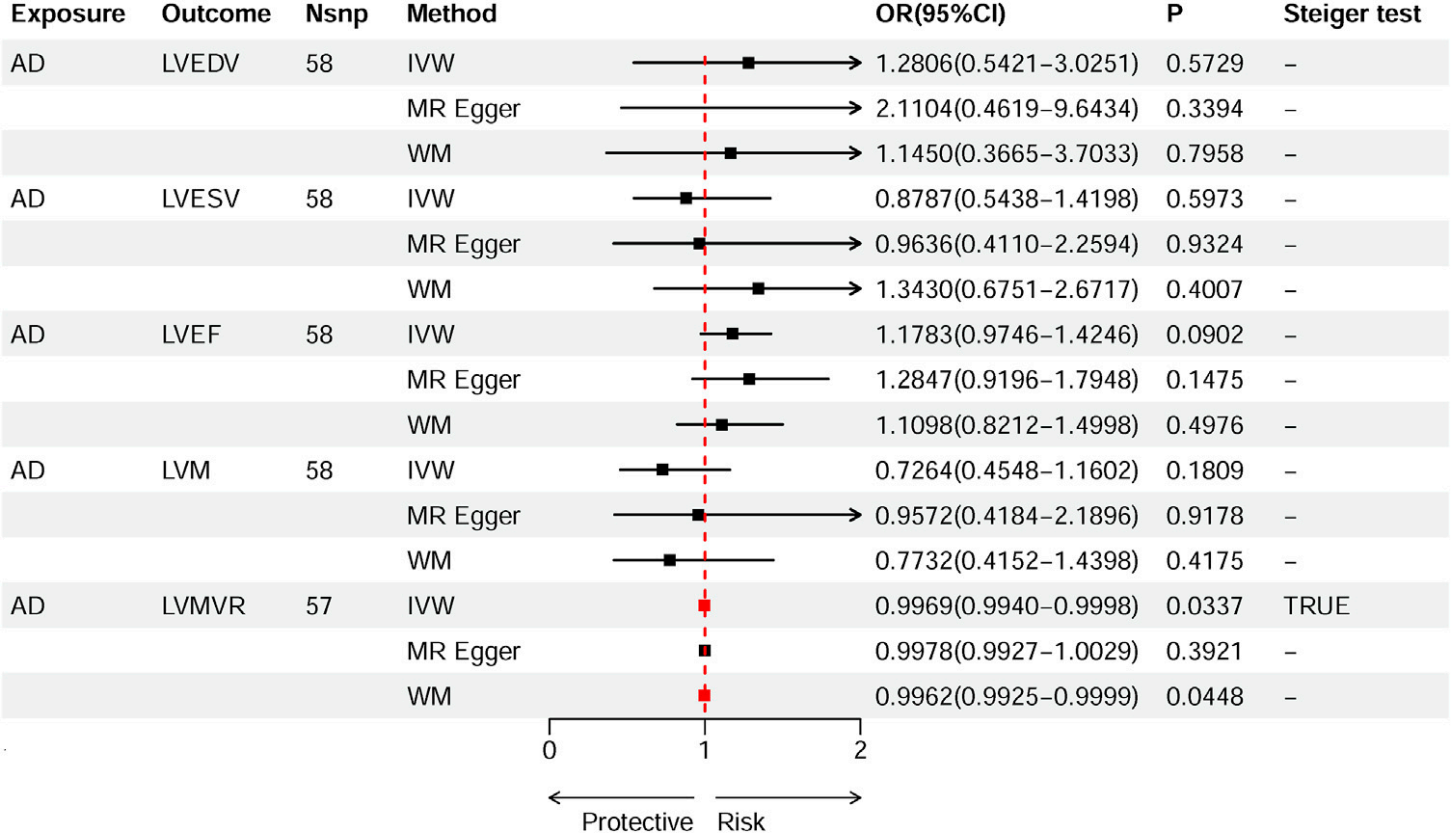
Causal effects of AD on left ventricular (LV) traits. Summary of the Mendelian randomization (MR) estimates derived from the inverse variance weighted (IVW), weighted median (WM), and MR-Egger methods. LVEDV, LV end-diastolic volume; LVESV, LV end-systolic volume; LVEF, LV ejection fraction; LVM, mass; LVMVR, left ventricular mass-to-end-diastolic volume ratio.

**FIGURE 5:**
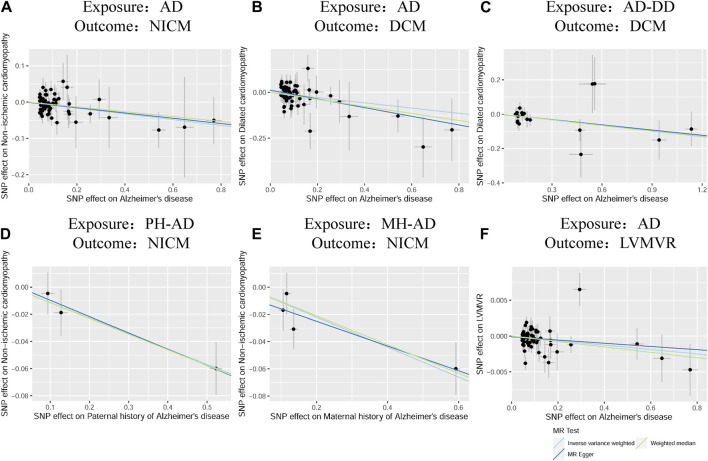
Scatter plots of the effect of AD on NICM, DCM, and left ventricular mass-to-end-diastolic volume ratio and scatter plots for the effect of MH-AD on NICM **(A–F)**. AD, Alzheimer’s disease; AD-DD, different dataset for Alzheimer’s disease; DCM, dilated cardiomyopathy; HCM, hypertrophic cardiomyopathy; LVMVR, left ventricular mass-to-end-diastolic volume ratio MH-AD, maternal history of Alzheimer’s disease; NICM, non-ischemic cardiomyopathy; PH-AD, paternal history of Alzheimer’s disease.

Based on IVW estimates, genetically predicted AD was nominally associated with decreased LVMVR (OR 0.9969, 95% CI 0.9940–0.9998, *p* = 0.0337). MR-Egger method (OR 0.9978, 95% CI 0.9927–1.0029, *p* = 0.3921) and WM method (OR 0.9962, 95% CI 0.9925–0.9999, *p* = 0.0448) showed consistent direction of the results (Figure 4). No causal effect of genetically predicted AD was found on the remaining four LV traits: LVEDV (OR 1.2806, 95% CI 0.5421–3.0251, *p-*_*IVW*_ = 0.5729), LVESV (OR 0.8787, 95% CI 0.5438–1.4198, *p-*_*IVW*_ = 0.5973), LVM (OR 0.7264, 95% CI 0.4548–1.1602, *p-*_*IVW*_ = 0.1809), and LVEF (OR 1.1783, 95% CI 0.9746–1.4246, *p-*_*IVW*_ = 0.0902). The scatter plots for the effect of AD on LVMVR are shown in Figure 5. The details can be obtained in Supplementary Table S8.

#### 3.2.4 Sensitivity analyses

A series of sensitivity analyses were performed to confirm the robustness of the significant IVW estimates between exposures and outcomes, whether nominal or not (Table 2). The Cochran’s Q test and MR-Egger intercept showed no evidence of heterogeneity and unbalanced directional pleiotropy (all *p*-values >0.05). The MR-PRESSO global test did not detect any evidence of pleiotropic effects (*p* > 0.05) for most of the causal estimates. Due to the deficiency of SNPs (3) derived from a causal estimate of PH-AD on NICM, it was not available for the MR-PRESSO test. The funnel plots were symmetrical, and the leave-one-out analysis indicated that the causal estimates were not biased by any of the SNPs (Supplementary Figures S1–S12). The results of the Steiger test between exposures and outcomes were all TRUE, suggesting no inverse causal link (Figures 2–4).

**TABLE 2:** Evaluation of heterogeneity and pleiotropy using different methods.

		IVW		MR egger		MR-PRESSO global test	MR-PRESSO distortion test
Exposure	Outcome	Q	*p*	Intercept	*p*	*p*	*p*
AD	NICM	53.2961	0.5400	−8.4437e-05	0.9852	0.551	NA
AD-DD	NICM	19.6228	0.2940	0.0047	0.5227	—	—
AD	DCM	58.2838	0.4648	0.0110	0.2598	0.486	NA
AD-DD	DCM	13.3302	0.7138	−0.0011	0.9378	0.735	NA
AD	HCM	61.2673	0.2613	−0.0292	0.1398	—	—
AD-DD	HCM	21.8000	0.1925	−0.0150	0.6347	—	—
MH-AD	NICM	1.6367	0.6511	−0.0069	0.6240	0.693	NA
MH-AD	DCM	12.4328	0.0060	−0.0271	0.7196	—	—
MH-AD	HCM	2.1780	0.5362	−0.0315	0.5873	—	—
PH-AD	NICM	0.2120	0.8995	0.0023	0.9048	NA	NA
PH-AD	DCM	5.9649	0.0507	0.0300	0.7645	—	—
PH-AD	HCM	4.4831	0.1063	−0.0486	0.7632	—	—
AD	LVMVR	71.8977	0.0747	−0.0001	0.680,298	0.087	NA

AD, Alzheimer’s disease; AD-DD, Different dataset for Alzheimer’s disease; DCM, dilated cardiomyopathy; GWAS, genome-wide association study; HCM, hypertrophic cardiomyopathy; IVW, inverse variance weighted; LVMVR, left ventricular mass-to-end-diastolic volume ratio, also known as left ventricular (LV) remodeling index; MH-AD, maternal history of Alzheimer’s disease; MR, Mendelian randomization; MR-PRESSO, MR pleiotropy residual sum and outlier; NA, not available; NICM, non-ischemic cardiomyopathy; PH-AD, paternal history of Alzheimer’s disease; Q, Q statistic.

## 4 Discussion

This was the first two-sample MR analysis that systematically evaluated the potential causal effects of AD and FHAD on NICM and LV structure and function. We identified five main associations with evidence of causality, one of which showed a strong causal effect after multiple corrections. Before correction, AD was significantly associated with a lower risk of NICM and DCM, and PH-AD and MH-AD were significantly associated with a lower risk of NICM. Despite the small effect size (OR: 0.9969), AD was nominally associated with a decreased LVMVR. After multiple corrections, we found a strongly significant association between MH-AD and a lower risk of NICM, with a *p*-value smaller than 7.82 × 10^−4^. Sensitivity analyses, including tests of heterogeneity and pleiotropy, the leave-one-out test, and the Steiger test, confirmed the robustness of our findings.

Misfolded proteins play a crucial role in the pathogenesis of neurodegenerative diseases. Previous reviews have elucidated the link between AD and cardiac dysfunction from the perspective of proteotoxicity to cardiomyocytes (Willis and Patterson, 2013). Proteotoxicity refers to the process by which protein aggregates formed by misfolded proteins can lead to cell death. Misfolded soluble protein oligomers can disrupt cellular function through multiple pathways, and this process is not limited to neurodegenerative diseases (Sanbe et al., 2004). Cardiomyocytes are composed of highly specialized components, forming a syncytium with a unified contractile function. Their function depends on protein homeostasis, including protein synthesis, folding, and turnover. The ubiquitin-proteasome system and autophagy are responsible for the turnover of misfolded proteins (Willis and Patterson, 2013). Oligomeric protein deposits in cardiomyocytes are important factors contributing to pathologic cardiac hypertrophy and DCM. For example, soluble oligomers are detected in cardiomyocytes of patients with HCM and idiopathic DCM but not in healthy controls (Sanbe et al., 2004). Cytosolic aggregates immunoreactive for ubiquitin have also been found in cardiomyocytes from the hearts of patients with DCM (Kostin et al., 2003). On the other hand, similarities in preamyloid oligomers were found in αB-crystallin (CryAB)-mutation-associated cardiomyopathy and AD (Maloyan et al., 2007). CryAB is expressed in the heart and can function as part of larger molecular complexes containing proteins that need to go through the folding process (Fu and Liang, 2003; Kampinga et al., 2009). Moreover, voluntary exercise is beneficial for AD associated with misfolded proteins. Decreased deposition of cardiac amyloid oligomers was observed when CryAB transgenic mice exercised voluntarily (Maloyan et al., 2007). A small-sized case-control study including participants without cardiac or systemic diseases found that compared to the control group, patients with AD had greater interventricular septum, greater maximum wall thickness, and a 2-fold higher prevalence of diastolic dysfunction in echocardiography (Sanna et al., 2019). The proteotoxicity hypothesis extends the link between AD and NICM and provides a new genetic perspective on the pathogenesis of NICM. However, our findings suggest that AD may reduce the risk of NICM, which encourages us to investigate the possible mechanisms behind this phenomenon from another perspective.

The brain-heart syndrome refers to the functional damage to the heart caused by brain disorders. Clinical and experimental studies have suggested that brain injuries, such as stroke (ischemic or hemorrhagic) and traumatic brain injury can lead to cardiac dysfunction and arrhythmias (Chen et al., 2017). Takotsubo cardiomyopathy, also known as stress cardiomyopathy, is characterized by transient left ventricular apical ballooning and is named after the Japanese octopus catcher pot (takotsubo) due to its similarity to the diseased heart (Tsuchihashi et al., 2001; Yoshimura et al., 2008). In patients with stroke, insular damage is a prominent predictor of takotsubo cardiomyopathy (Yoshimura et al., 2008). Decreased heart rate variability (HRV) has been observed in patients with an insular infarct, particularly among those with a right side infarct (Tokgözoglu et al., 1999). The autonomic nervous system consists of various interconnected areas in the telencephalon, diencephalon, and brainstem, including the insular cortex and hypothalamus (Wehrwein et al., 2016). The insular cortex appears to play a leading role in autonomic control of cardiac activity. The hypothalamus, locus coeruleus, cerebral neocortex, insular cortex, and brainstem related to the control of sympathetic and parasympathetic outflow are affected in AD. In addition, autonomic dysfunction in AD can be assessed by calculating HRV, which is closely related to cognitive dysfunction (Braak and Braak, 1995; Femminella et al., 2014; Giraldo et al., 2018). It can be inferred that central cholinergic malfunctioning may lead to autonomic dysfunction, establishing a link between higher cerebral and autonomic neural functions. Autonomic dysfunction of the heart was assessed by HRV in patients with AD, which was significantly correlated with the plasma activity of acetylcholinesterase (Giubilei et al., 1998). DCM is associated with decreased parasympathetic activity and enhanced sympathetic activity (Ajiki et al., 1993). Observational studies have also found a link between HRV and DCM (Hoffmann et al., 2000; Rashba et al., 2006; Grutter et al., 2012). Specifically, HRV values were significantly reduced in adult patients with DCM (Yi et al., 1997) and increased in male children with DCM who were less than 6 years of age (Grutter et al., 2012). In addition, adult patients with DCM and preserved HRV had a better prognosis (Yi et al., 1997; Rashba et al., 2006). However, HRV cannot help determine the type of autonomic dysfunction in patients with AD (inhibition or hyperfunction) (Mellingsæter et al., 2015; Belaidi and Bush, 2016; Santos et al., 2017). Therefore, we believe that HRV alone is insufficient to assess autonomic dysfunction in AD. Furthermore, we found that changes in HRV (reduced or increased) are opposite in patients with DCM at different ages (less than 6 years old or not) (Yi et al., 1997; Grutter et al., 2012), suggesting that different stages of AD (mild or severe) may have different effects on cardiac autonomic function.

Similarly, we considered stress cardiomyopathy as a result of neurological injury to understand the role of the sympathetic nervous system in the brain-heart interaction and attempted to link altered sympathetic function to AD. The hypothalamic paraventricular nucleus (HPN) controls sympathetic activity, while higher brain regions regulate sympathetic outflow to the heart through the HPA axis (Chen et al., 2017). Activation of the sympathetic nervous system can induce arrhythmias and myocardial necrosis when the hypothalamus is stimulated (Melville et al., 1963). In response to stress stimuli, the HPN secretes corticotropin-releasing factor that stimulates the pituitary gland to release adrenocorticotropic hormone, thereby increasing cortisol levels (Whitnall, 1993). Cortisol levels are related to stroke severity (Christensen et al., 2004). It has been found that during stressful conditions, structural and functional changes occur in brain regions associated with cognitive functions (Lupien et al., 2009). Additionally, higher cortisol levels in the cerebrospinal fluid (CSF) have been observed in patients with mild cognitive impairment compared to cognitively healthy populations (Ouanes and Popp, 2019). The catecholamine surge is widely believed to induce cardiac dysfunction after ischemic stroke. Increased sympathetic tone after brain injury manifests with a catecholamine surge. High levels of catecholamines led to cardiac hypertrophy or myocardial ischemia in animal experiments (van der Bilt et al., 2016; Chen et al., 2017). Elevated catecholamine levels have also been observed in patients with AD (Janssens et al., 2019). However, CSF or plasma levels of catecholamines dynamically change with AD progression (Janssens et al., 2019; Pillet et al., 2020; Henjum et al., 2022). Higher CSF levels of noradrenaline and adrenaline were found in patients in the early stages of AD (Henjum et al., 2022), but the CSF levels of norepinephrine decreased in more severe cases (Janssens et al., 2018). A similar trend was observed in the plasma concentrations of norepinephrine, which were significantly lower in control subjects and patients with advanced AD than in patients with early-stage AD (Pillet et al., 2020). Meanwhile, neuronal loss and shrinkage of the locus coeruleus (the primary site of noradrenaline synthesis) occur during AD progression (Takahashi et al., 2015; Kelly et al., 2017). We hypothesized that differences in the plasma levels of norepinephrine may be related to blood-brain barrier breakdown and locus coeruleus shrinkage, which weakens the central control of the sympathetic system. Autonomic failure is often characterized by reduced sympathetic outflow and is associated with neurodegenerative diseases. Compared to autonomic failure, sympathetic storms occur after catastrophic neurological injuries, and stress-induced cardiomyopathy arises after exposure to a catecholamine surge (Tahsili-Fahadan and Geocadin, 2017). As the disease progresses, autonomic failure may exceed sympathetic activation, leading to decreased norepinephrine levels in patients with advanced AD (Janssens et al., 2019). Based on these findings, we suggest that autonomic failure may explain how AD protects against NICM. Further studies are needed to confirm that autonomic failure characterized by decreased norepinephrine levels occurs in patients with advanced AD.

Risk factors and protective factors associated with AD also affect NICM. Recently, Ewers et al. reported that soluble triggering receptor expressed on myeloid cell 2 (TREM2) is associated with milder cognitive decline in patients with AD (Ewers et al., 2019). The CSF concentrations of soluble TREM2 were found to increase with neurodegeneration and potentially reflect microglial activity. Microglia activated by the TREM2 signaling show greater chemotaxis and phagocytosis and may regulate the progression of amyloid plaques (Keren-Shaul et al., 2017; Krasemann et al., 2017). Using single-cell RNA sequencing and fate mapping, Fang et al. identified a unique subset of heart-resident macrophages (CD163^+^RETNLA^+^) in a mouse model of sepsis. This subset exhibited high expression of TREM2 and actively removed dysfunctional mitochondria excreted by cardiomyocytes. Conversely, macrophages lacking *Trem2* were unable to self-renew, resulting in an excessive inflammatory response in cardiac tissue and more severe cardiac dysfunction (Zhang et al., 2023). ApoE was originally identified as a major ligand for low-density lipoprotein (LDL) and is involved in lipid metabolism. Increased levels of LDL, as a consequence of apoE2 and apoE4, increase the risk of heart disease. The fragments produced by structurally altered apoE4 undergoing neuron-specific proteolysis can lead to mitochondrial dysfunction and tau phosphorylation (Mahley, 2016). However, animal models of NICM have demonstrated that senile left ventricular hypertrophy and diastolic dysfunction develop in apoE-deficient mice in the absence of a high-fat diet, finally leading to heart failure (Liehn et al., 2021). Another cause of familial AD is the mutation of presenilin 1 (*PSEN1*) or presenilin 2 (*PSEN2*), which may be associated with DCM. Mutations of *PSEN1* and *PSEN2* have different effects on DCM. The former is associated with complete penetrance and advanced AD, whereas the latter exhibits partial penetrance and a better prognosis (Li et al., 2006).

LV dilation, systolic dysfunction, and decreased LVEF are the key features of DCM, making the assessment of LV size and LVEF crucial to the diagnosis, risk stratification, and treatment of DCM (Japp et al., 2016; Merlo et al., 2018). Ventricular remodeling is a major mechanism driving the progression of DCM to heart failure (Tobita et al., 2018). In this study, we investigated the causal effects of AD on five parameters representing the structure and function of the LV and found that AD was associated with decreased LVMVR (OR 0.9969, *p-*_*IVW*_ = 0.0337). This finding is consistent with prior results regarding the protective effect of AD on NICM/DCM. LVMVR (also known as LV remodeling index) is a strong and independent predictor of diastolic function in patients with DCM (Buakhamsri et al., 2009; Xie et al., 2020). LVMVR has been shown to increase with age in both men and women (Peverill, 2021). A recent study demonstrated that higher LVMVR was significantly correlated with reduced LVEF in patients with type 2 diabetes (Xie et al., 2020). Although the effect size was small (OR: 0.9969), genetically predicted AD showed a negative effect on LVMVR, further supporting the reliability of the causal effect of AD on NICM/DCM.

Genetic factors play a significant role in both AD and DCM (Weintraub et al., 2017; Scheltens et al., 2021). In addition, we measured the causal effect of family history on NICM and found the substantial effect of genetic factors on the incidence of AD. Using MR analysis, we investigated the causal association between AD and NICM/DCM, providing a deeper insight into the involvement of genetic factors in the pathogenesis of NICM. Consistent with the results of MR, AD and DCM are genetically heterogeneous and cannot be solely attributed to individual DNA variants with large effects. Many variants with small effects on disease risk can result in noticeable phenotypes, and genetic or environmental modifications are increasingly important. Therefore, examination of more complex genetic factors can provide a deeper insight (Sierksma et al., 2020; Tayal et al., 2021).

We used GWAS summary statistics and MR analysis to investigate the causal effect of AD and FHAD on NICM/DCM and measure the causal effect of AD on LV traits. We addressed a novel issue that was not previously explored. Another advantage of this study was the use of MR analysis, which reduces potential biases caused by confounding factors and prevents reverse causation, commonly observed in traditional observational studies. Sensitivity analyses, including identification of heterogeneity and pleiotropy, leave-one-out analysis, and MR Steiger directionality test, were conducted to verify the robustness of our results. However, all participants were of European origin. Although it reduces the potential bias associated with demographics, it limits the generalizability of MR results to other populations. More importantly, although MR analysis suggested that AD may protect against NICM, further mechanistic and experimental studies are needed to confirm the genetic associations. The involvement of autonomic failure in the protective effect of AD on NICM is only a hypothesis based on the course of AD; thus, we need to treat this causality with caution.

## 5 Conclusion

In conclusion, our study uncovered the causal effects of AD on NICM/DCM and LV traits and revealed that AD and FHAD were associated with a decreased risk of NICM. Our findings implied that AD may be useful in etiological studies for NICM. Further studies are warranted to illuminate the underlying mechanisms linking AD to NICM.

## Data Availability

The data presented in the study are deposited in https://www.ebi.ac.uk/gwas/, accession number (PMID): 35379992/30820047/29777097/34594039/33495596; and https://gwas.mrcieu.ac.uk/, accession number (GWAS ID): finn-b-I9_NONISCHCARDMYOP/finn-b-I9_HYPERTROCARDMYOP.

## Abbreviations

AD, Alzheimer’s disease; AD-DD, Different dataset for Alzheimer’s disease; apoE, apolipoprotein E; DCM, dilated cardiomyopathy; FHAD, family history of Alzheimer’s disease; GWAS, genome-wide association study; HCM, hypertrophic cardiomyopathy; LV, left ventricular; LVEDV, LV end-diastolic volume; LVESV, LV end-systolic volume; LVEF, LV ejection fraction; LVM, mass; LVMVR, left ventricular mass-to-end-diastolic volume ratio; MH-AD, maternal history of Alzheimer’s disease; MR, Mendelian randomization; MR-PRESSO, MR pleiotropy residual sum and outlier; NA, Not Applicable; NICM, non-ischemic cardiomyopathy; PH-AD, Paternal history of Alzheimer’s disease; SNPs, single nucleotide polymorphisms.
