# Supplementary material for: The causal effect of Alzheimer’s disease and family history of Alzheimer’s disease on non-ischemic cardiomyopathy and left ventricular structure and function: a Mendelian randomization study
Source: Front Genet. 2024 Jun 5;15:1379865. doi: 10.3389/fgene.2024.1379865 (PMC11188370; doi:10.3389/fgene.2024.1379865)
Supplement: Supplementary file 2 [file Table1.DOCX]

Figure S1. Funnel plot for Alzheimer's disease on non-ischemic cardiomyopathy.

Figure S2. Funnel plot for Alzheimer's disease on dilated cardiomyopathy.

Figure S3. Funnel plot for Alzheimer's disease (different dataset) on dilated cardiomyopathy.

Figure S4. Funnel plot for paternal history of Alzheimer's disease on non-ischemic cardiomyopathy.

Figure S5. Funnel plot for maternal history of Alzheimer's disease on non-ischemic cardiomyopathy.

Figure S6. Funnel plot for Alzheimer's disease on left ventricular mass-to-end-diastolic volume ratio (LVMVR).

Figure S7. Leave-one-out analysis for Alzheimer's disease on non-ischemic cardiomyopathy.

Figure S8. Leave-one-out analysis for Alzheimer's disease on dilated cardiomyopathy.

Figure S9. Leave-one-out analysis for Alzheimer's disease (different dataset) on dilated cardiomyopathy.

Figure S10. Leave-one-out analysis for paternal history of Alzheimer's disease on non-ischemic cardiomyopathy.

Figure S11. Leave-one-out analysis for maternal history of Alzheimer's disease on non-ischemic cardiomyopathy.

Figure S12. Leave-one-out analysis for Alzheimer's disease on LVMVR.


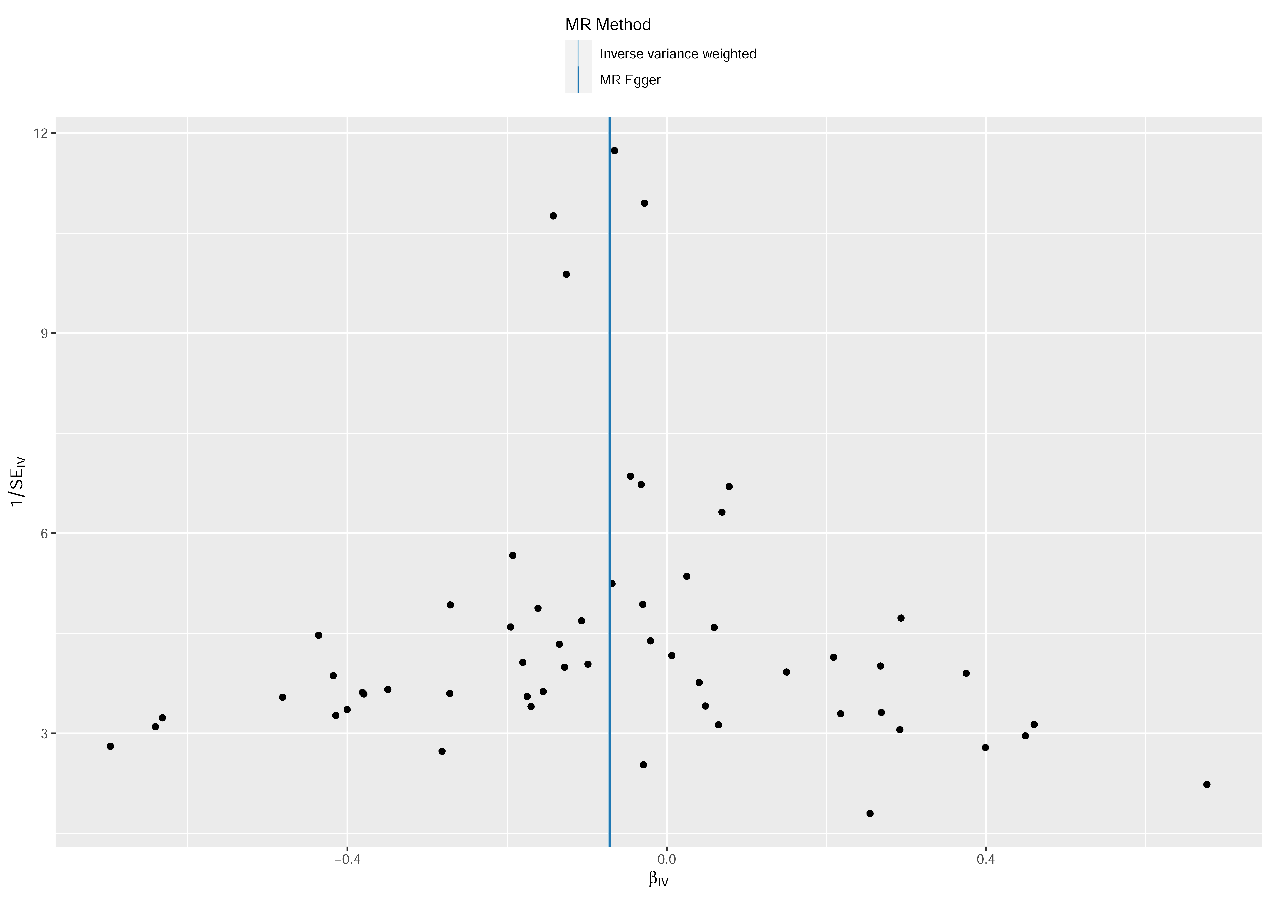


Figure S1. Funnel plot for Alzheimer's disease on non-ischemic cardiomyopathy.


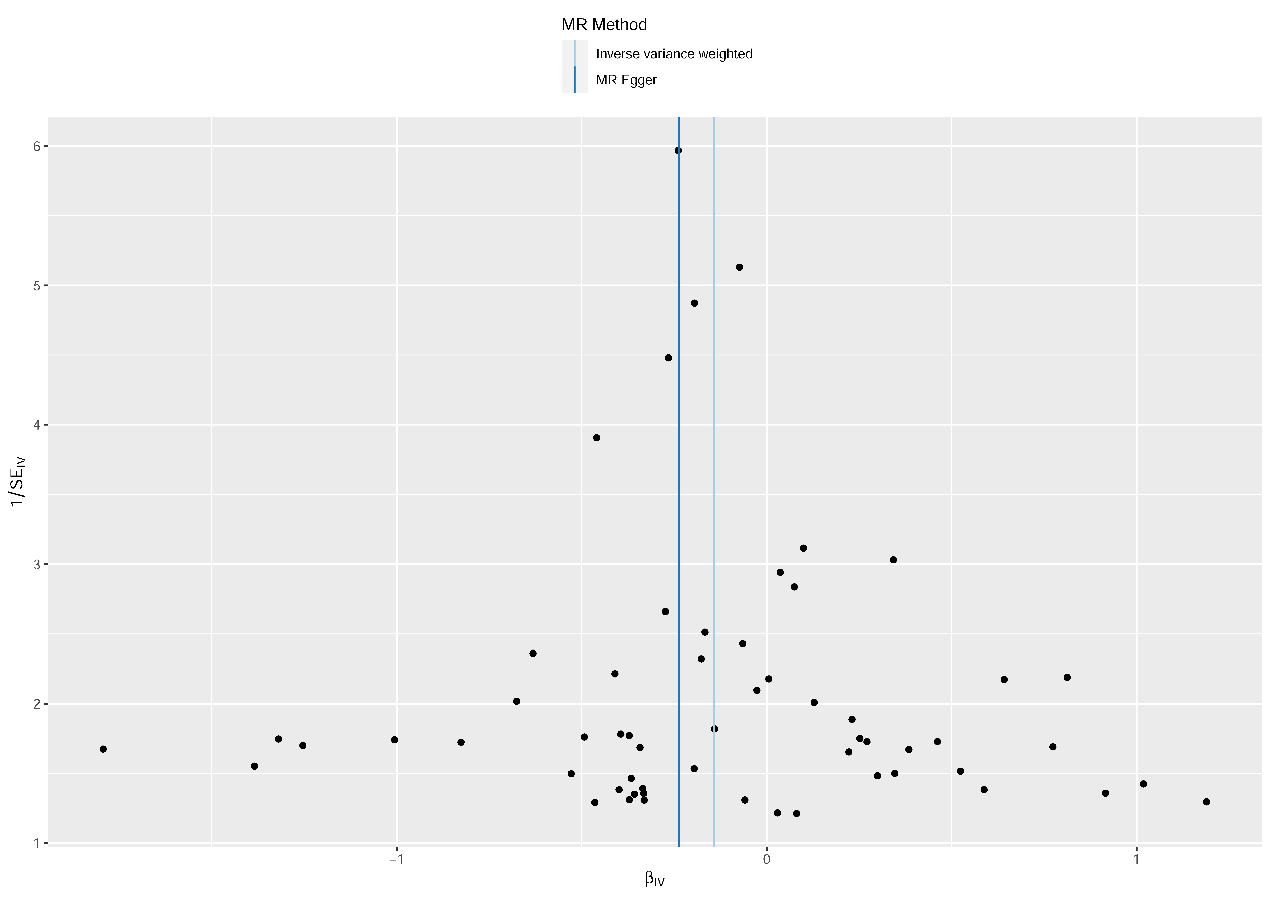


Figure S2. Funnel plot for Alzheimer's disease on dilated cardiomyopathy


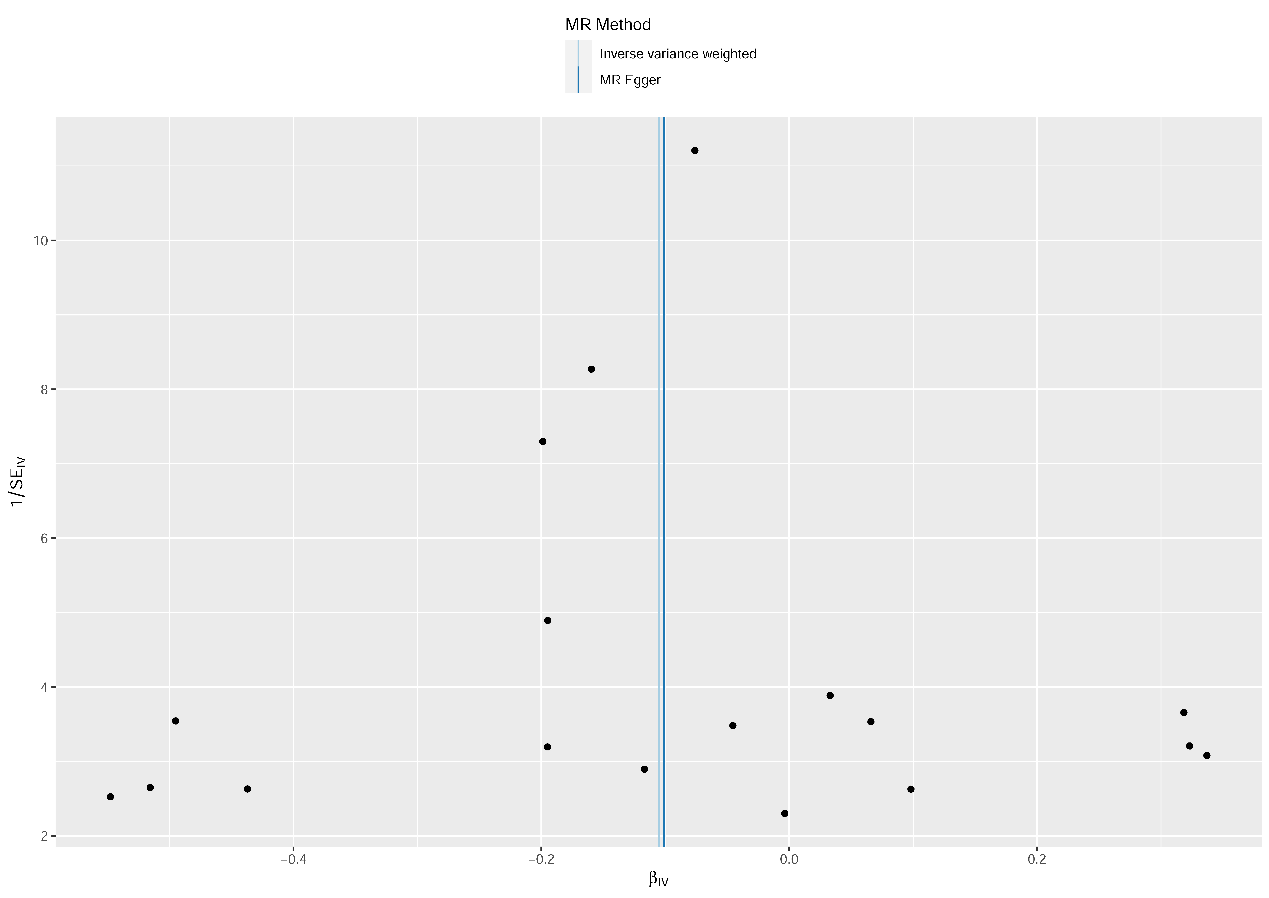


Figure S3. Funnel plot for Alzheimer's disease (different dataset) on dilated cardiomyopathy.


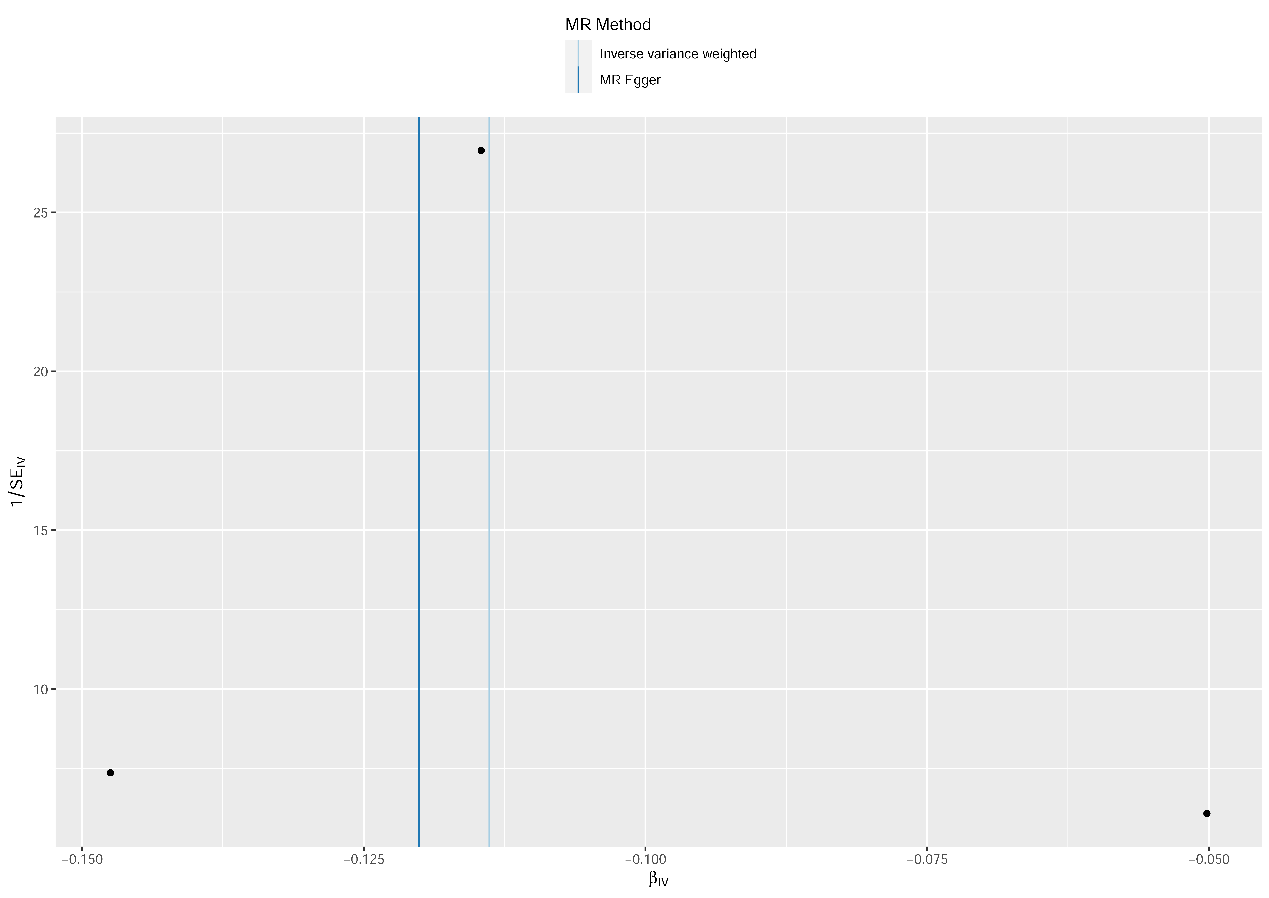


Figure S4. Funnel plot for paternal history of Alzheimer's disease on non-ischemic cardiomyopathy.


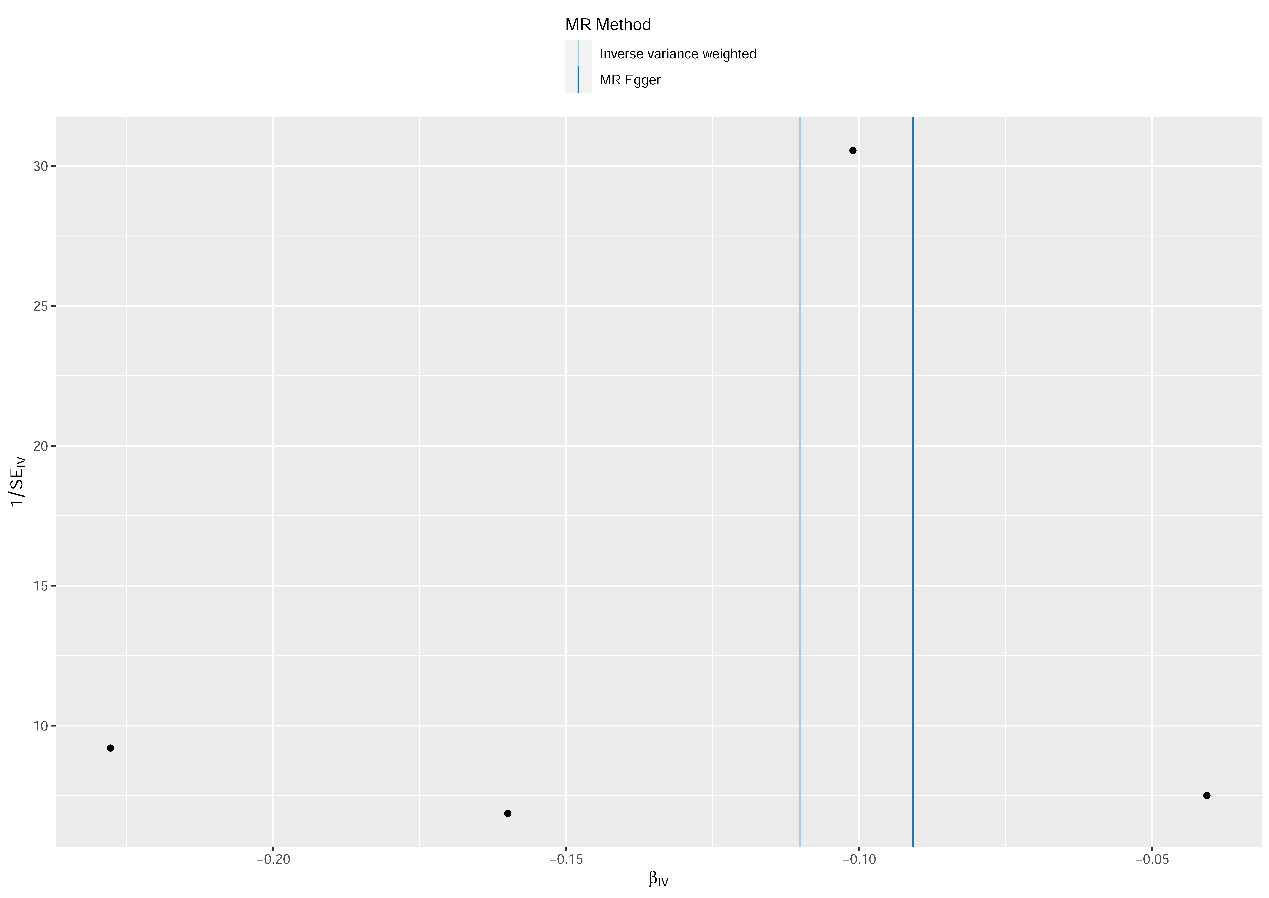


Figure S5. Funnel plot for maternal history of Alzheimer's disease on non-ischemic cardiomyopathy.


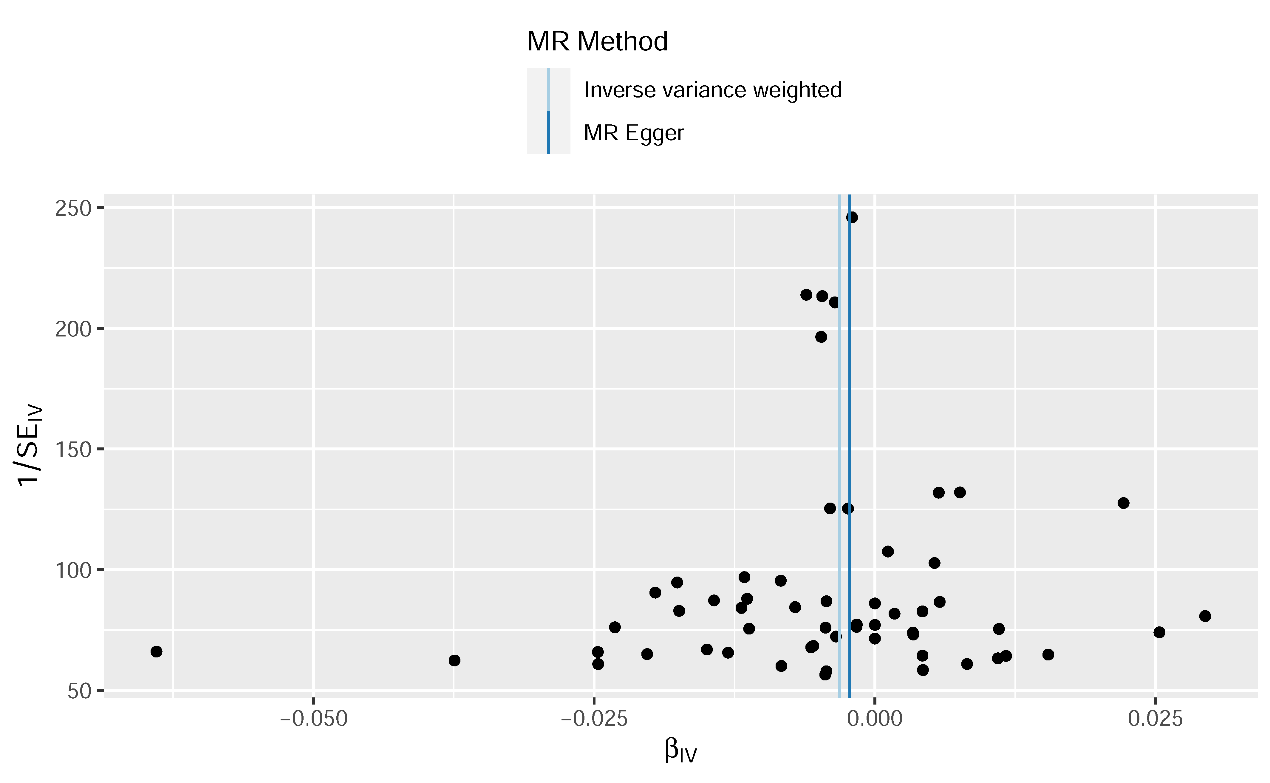


Figure S6. Funnel plot for Alzheimer's disease on left ventricular mass-to-end-diastolic volume ratio (LVMVR).


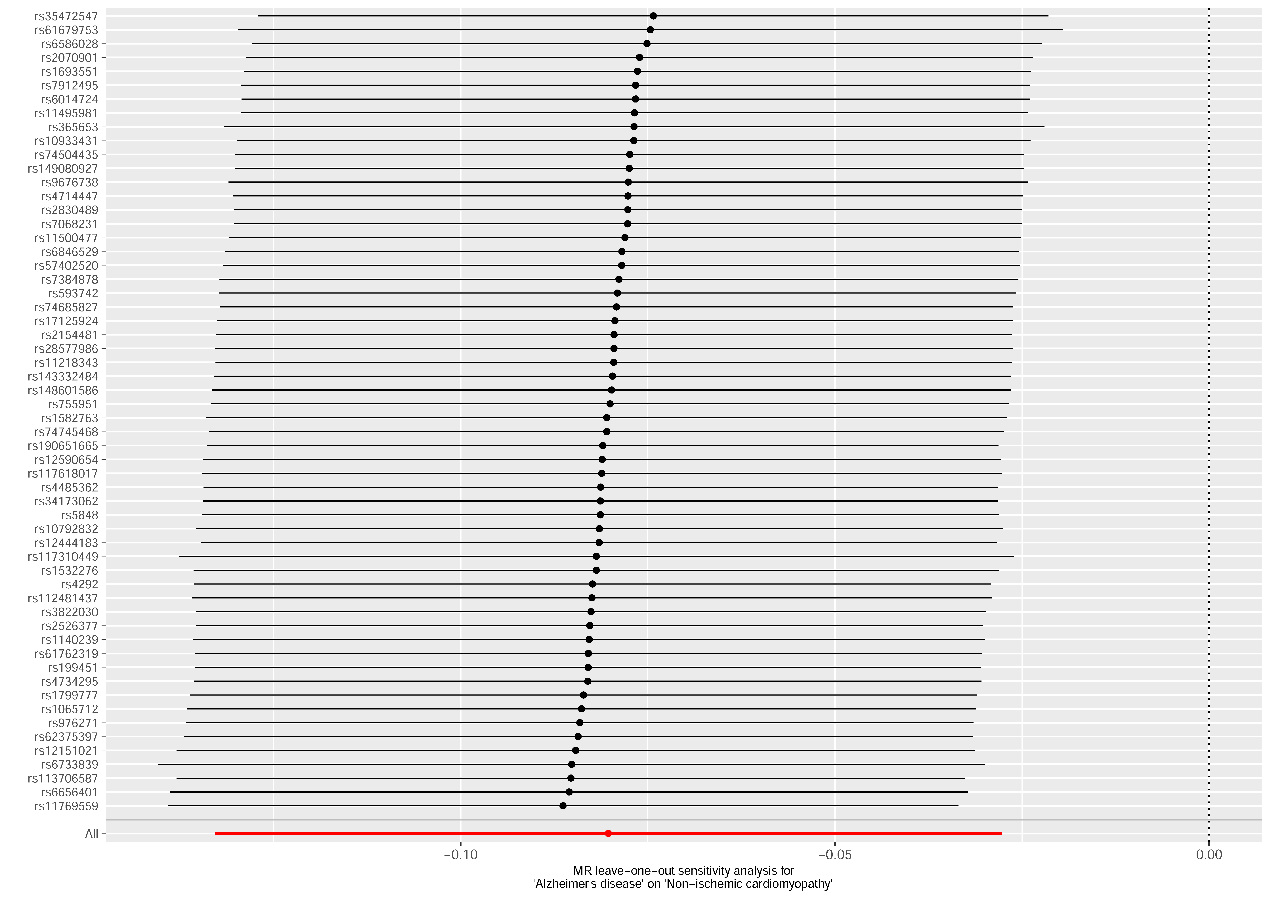


**Figure S7.** Leave-one-out analysis for Alzheimer's disease on non-ischemic cardiomyopathy.
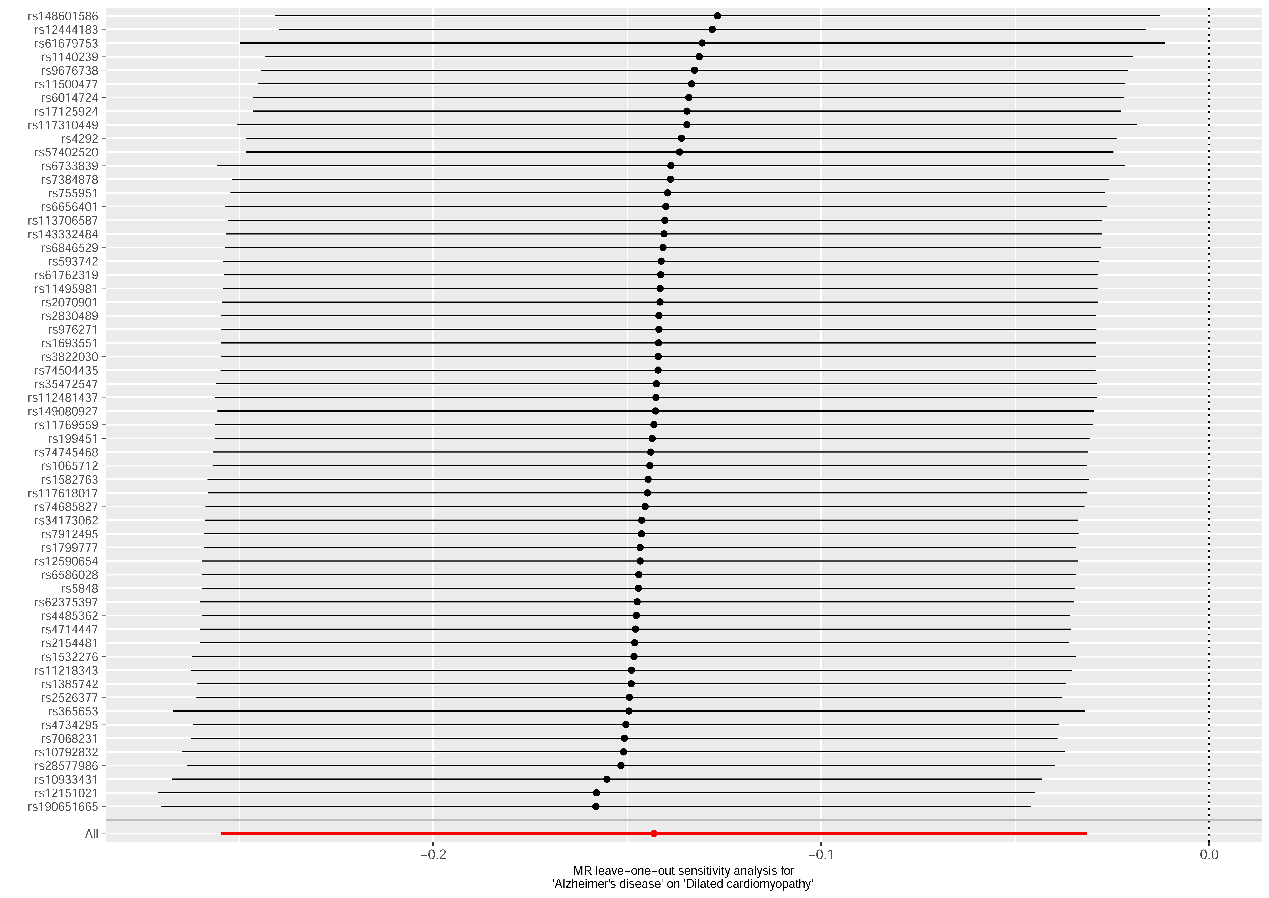


**Figure S8.** Leave-one-out analysis for Alzheimer's disease on dilated cardiomyopathy.
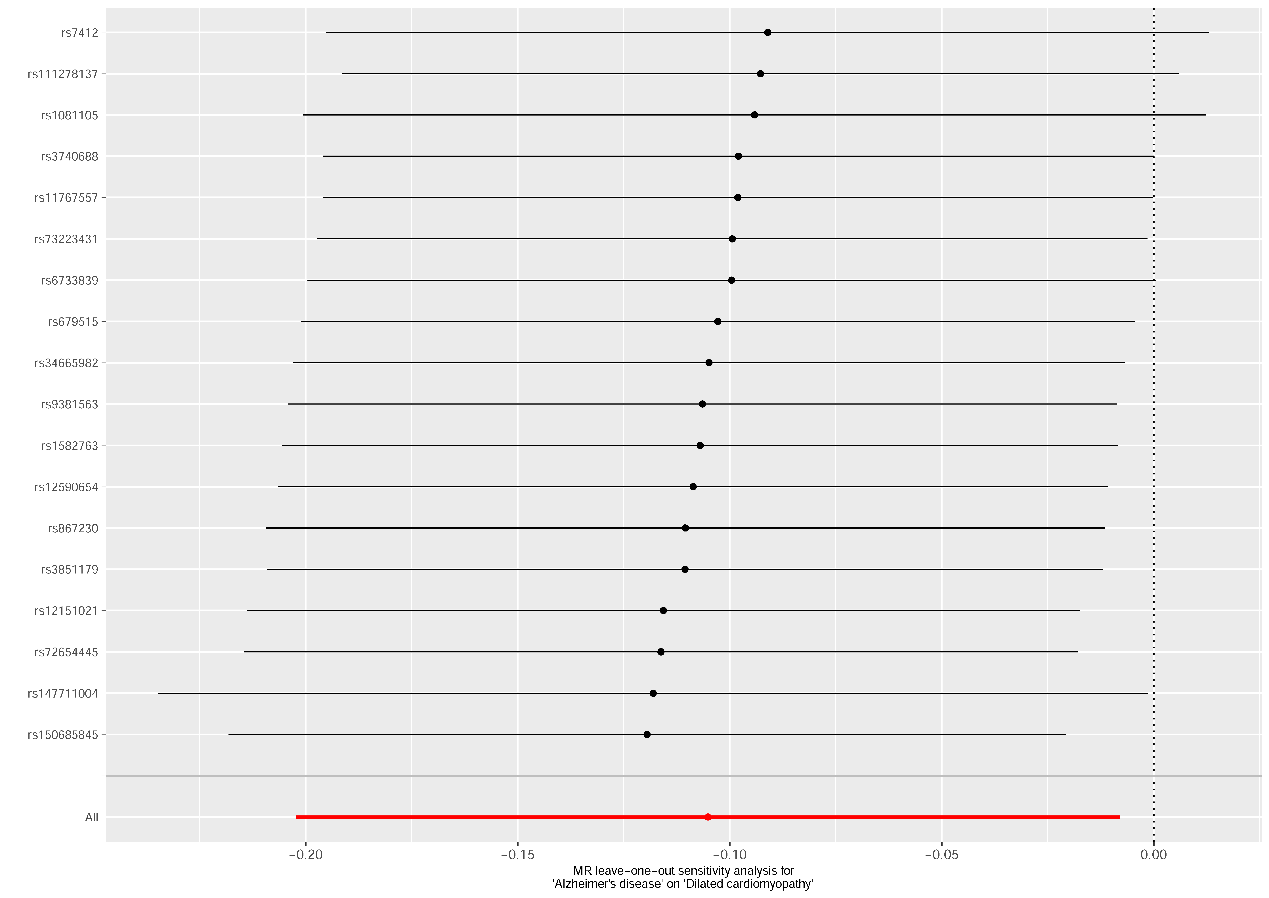


Figure S9. Leave-one-out analysis for Alzheimer's disease (different dataset) on dilated cardiomyopathy.


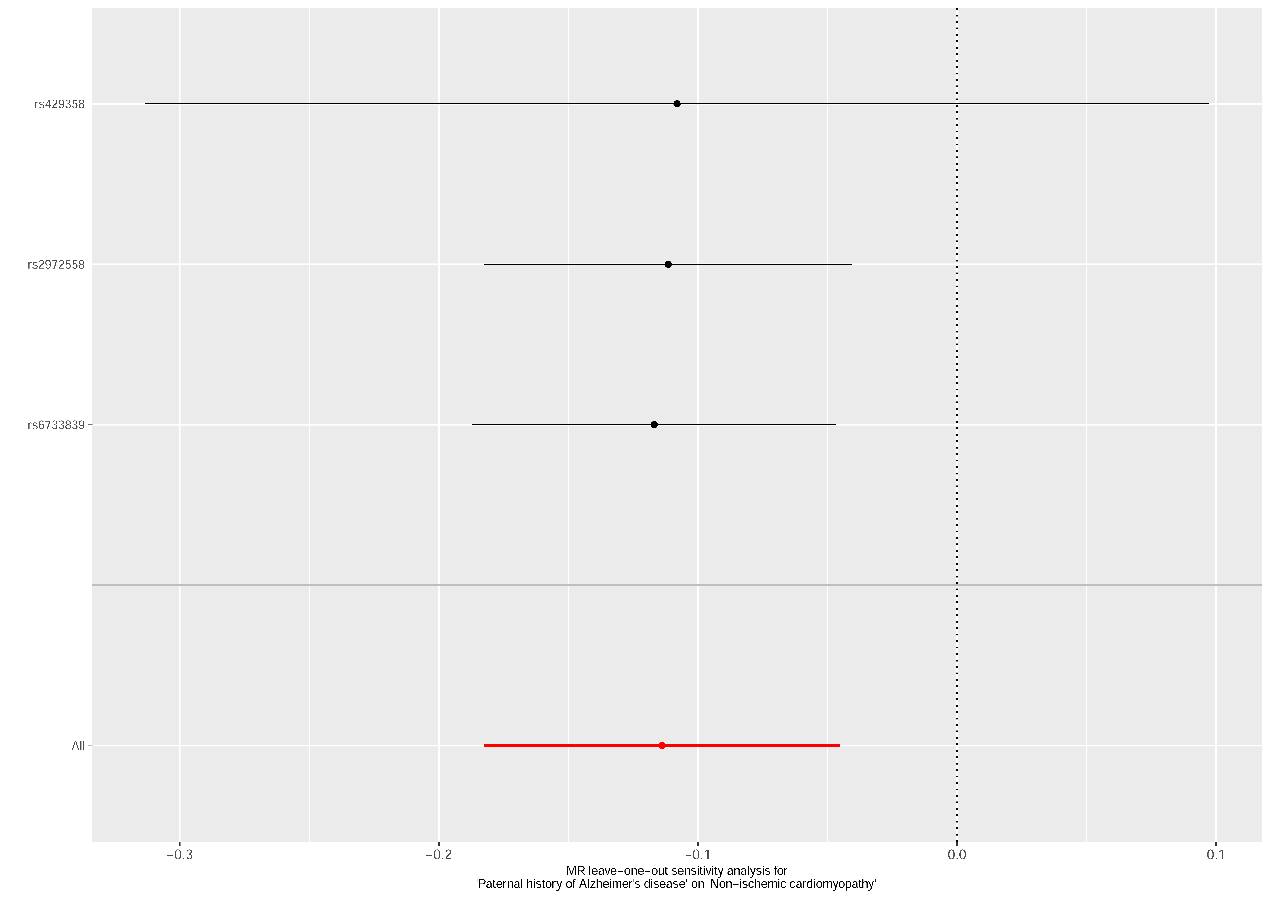


**Figure S10.** Leave-one-out analysis for paternal history of Alzheimer's disease on non-ischemic cardiomyopathy.


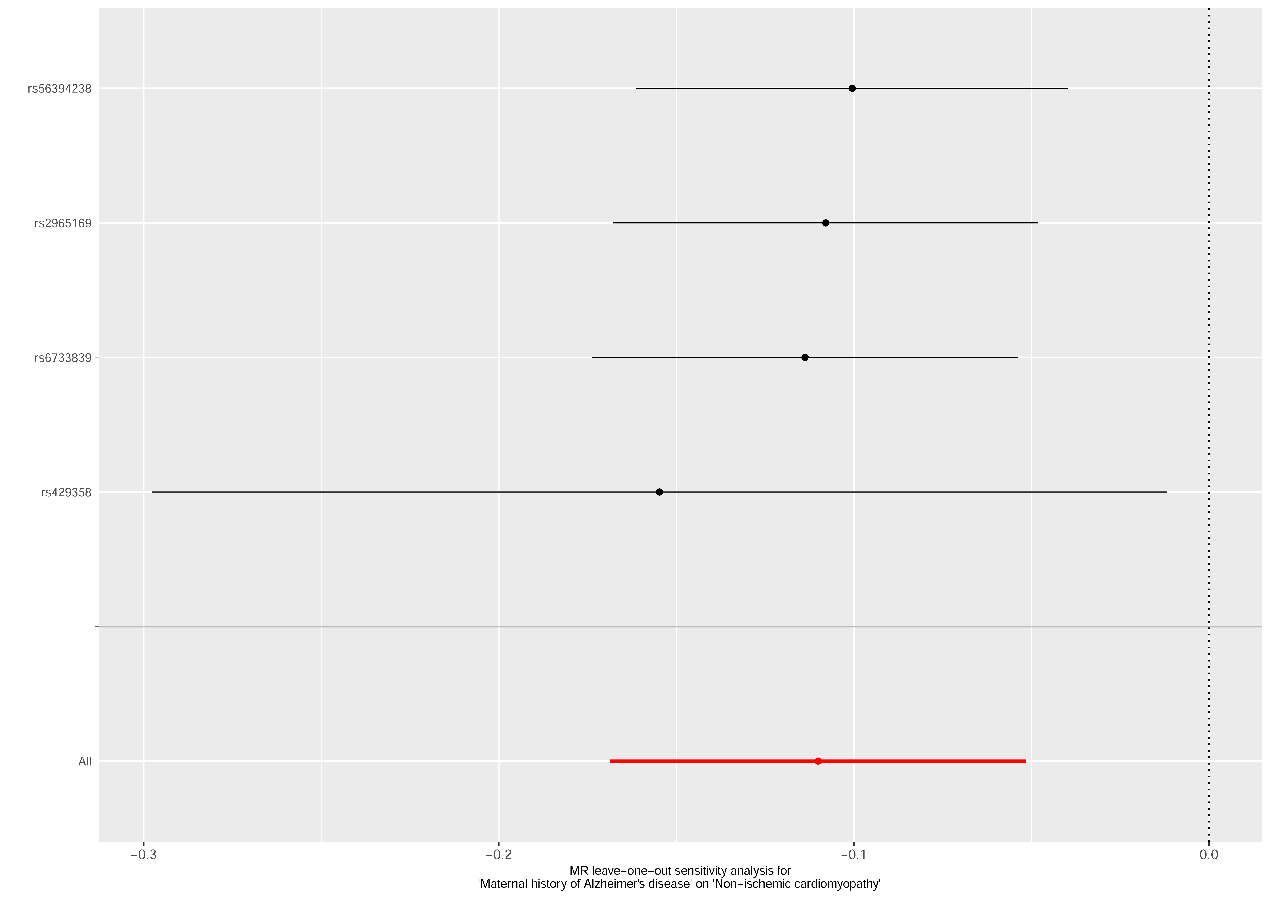


Figure S11. Leave-one-out analysis for maternal history of Alzheimer's disease on non-ischemic cardiomyopathy.


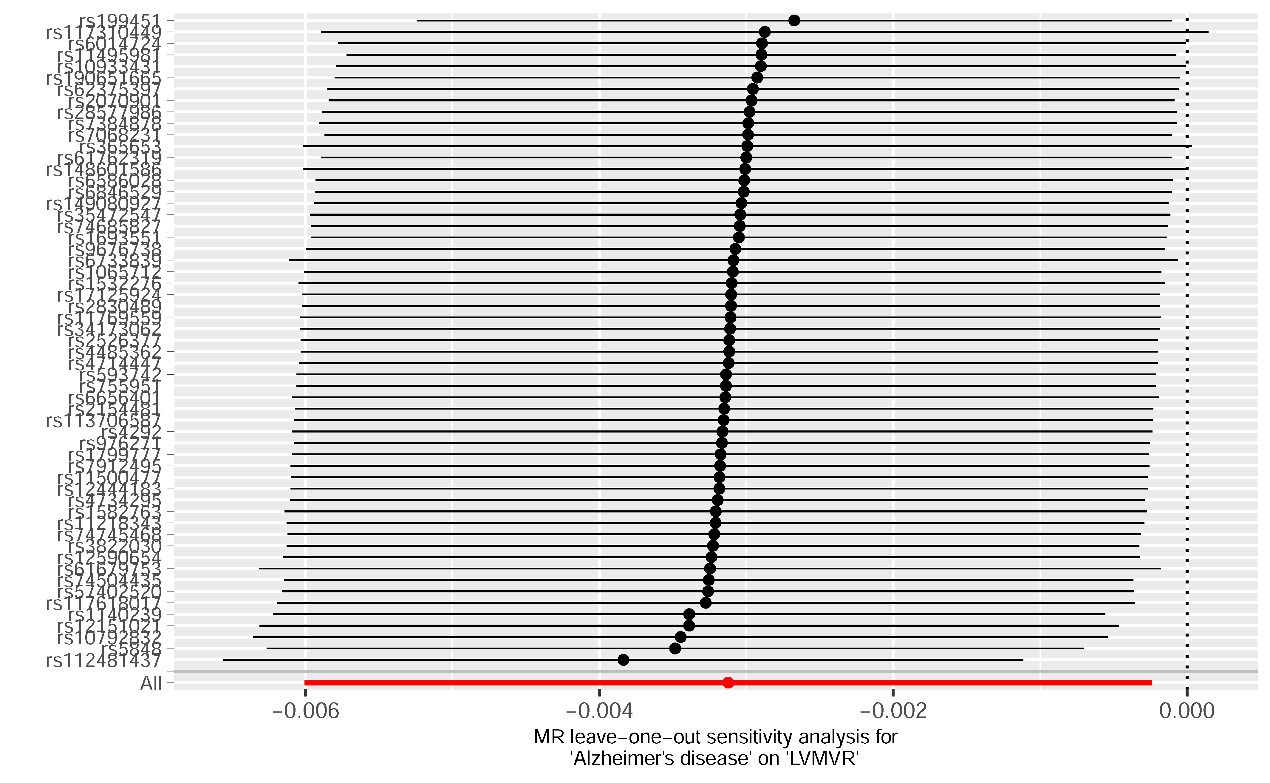


Figure S12. Leave-one-out analysis for Alzheimer's disease on LVMVR.
